# Practical Medicine

**Published:** 1877-11

**Authors:** 


### III. Practical Medicine.

Treatment of Neuralgia by Hypodermic Injections of Nitrate of Silver.—Le Dentu. (*La France Médic*, 1877.) —The author has recently reported to the Society of Clinical Medicine several cases treated successfully by the method first suggested by a M. Luton.

A young woman suffered for four months from a neuralgia of the radial nerve which had been treated in all possible ways. In view of this want of success, Le Dentu decided to inject subcutaneously in the cellulo-fatty tissue found in the middle third of the external face of the arm, seven drops of a solution of nitrate of silver (1 to 4). The result was that an intense pain succeeded immediately the pain of the neuralgia, the latter not once recurring after the moment in which the injection was made. On that and the succeeding days a very painful swelling invaded the arm and the upper part of the fore-arm with an œdematous and subsequently phlegmonous tumefaction at the site of the puncture. After nine or ten days an abundant sero-pus flowed from the latter, but the suppuration gradually diminished and the cure was finally complete. Two months afterward it was ascertained that the relief was absolute.

Having had since then no opportunity to make further trial in obstinate neuralgias of Luton's method, the author concluded to employ it in painful affections of another kind. A man who had been castrated on the right side and had been relieved by the operation, was tuberculous and suffered from frequent hæmaturia, violent pain in the glans penis and in the lower part of the belly and lumbar region, the permanent pain being aggravated during and immediately after micturition, but never entirely ceasing. The lumbar and testicular pain disappeared under the influence of punctate cauterizations, but the hypogastric neuralgia persisted. Le Dentu then made a subcutaneous injection into the hypogastric region, of four drops of a solution of the nitrate of silver (1 part to 5). Very intense pain resulted with phlegmon and suppuration on the fourth day. Quite thick pus escaped after an opening was made. But in nine days the patient was quite satisfied with the result, for although he still suffered from the pain during micturition, it had completely disappeared during the interval.

Again in a patient with scapulalgia, where punctate cauterization had effected no result, an injection of four drops of the same solution into the connective tissue immediately in front of the painful region, was followed by intense pain; local suppuration and an abscess on the fifth day. But on the second day after the injection the deep seated pain was completely relieved, and movement of the limb was readily effected.

Le Dentu concludes with the remark that he has always seen a small abscess result from the operation, but that by opening the former prematurely—say on the fourth or fifth day, this inconvenience would be much lessened.

Gastric and Intestinal Gases, with Flatulent Dyspepsia. —Leven. (*L' Union Méd.*, Oct. 11, 1877.) The following are the author's conclusions:

Alimentary substances do not seem to produce gas; that which is found in the digestive tube is derived from external air, blood or fecal matter. The gases produced in flatulent dyspepsia are not due to the decomposition of alimentary substances, but are derived from the three sources mentioned above. They are kept in continual motion by the pathological contraction of the muscular fibres of the intestine; they are continually renewed; their production may be incessant, as well in an individual who is fasting, as in one who is nourished.

This symptom, production of gas, signifies, therefore, an intestinal irritation which is always consecutive to a prior gastric dyspepsia.

The course of the disease and the treatment requisite to cure it, confirm these clinical facts.

There is no necessity for seeking a medication to relieve the gas, and, as for the powders, such as charcoal, which are said to absorb it, they actually have no such effect, as the author has proved by experiment. A block of charcoal, it is true, may absorb gas, but as soon as it is reduced to powder it loses its absorptive power.

A NEW INVESTIGATION OF THE RÔLE OF THE ALKALIES IN THE ANIMAL ECONOMY.—Mialhe. (*L'Union Méd.*, Oct. 11, 1877.) The fact of the indispensable necessity of the constant presence of the alkalies in the animal economy was first established, in 1824, by Chevreul; but he attributed this to a need of the caustic alkalies, while it should be properly referred to the alkaline bicarbonates.

In the present state of science we are authorized to say that the influence of the alkalies upon organic matters, which renders possible their oxydation in the sphere of the animal economy, can no longer be questioned. This the author has demonstrated in several memoirs on digestion, assimilation and organic or vital oxydation. Inorganic bodies certainly exist in the organs of animals, and these exhibit an indisputable activity; such are iron, phosphate of lime and the alkaline bicarbonates.

Resting upon these facts, Mialhe endeavors to solve the question: Can the alkaline bicarbonates, when administered in large doses, give rise to a special cachexia, designated as the alkaline cachexia? This question he answers in the negative, and concludes with some general considerations relative to the administration of the alkalies.

What is proposed by the administration of the sodic bicarbonated waters? The end desired is the introduction into the blood of a proportion of the bicarbonate of soda, sufficient to sensibly modify the intimate composition of the albuminoid materials with which the alkaline element enters into combination; and, as a result, the stimulation of the phenomena of organic or vital oxydation, as well as that of endosmosis and exosmosis, with a view to modify the nature of secretions, etc.

What, now, is the proportion of the bicarbonate of soda which it is proper to introduce into the economy, in order to produce this result?

It is impossible to give a categorical answer to this question, because the proportion of the alkaline base which exists in the animal economy, whether in the state of the bicarbonate or of the albuminate, is far from being always the same, not only in the animals of the two great classes, the herbivora and the carnivora, but also in animals of the same species. Thus the herbivora have their humors alkaline in a different sense from the carnivora, whence it follows that man, who is omnivorous, should occupy a middle ground between the two, and, from the point of view of his humoral alkalinity, approach either the herbivora or the carnivora, according to the nature of his alimentation. This is precisely what occurs. The inhabitant of the city, the wealthy individual, has need of a larger dose of alkali to restore his humors to a physiological state, than the person who lives almost exclusively upon a vegetable diet.

The function of the skin should not be neglected. The practitioner should remember, in prescribing alkalies, that a patient who perspires, loses a portion of his acids from his economy, and to be properly "alkalized" he requires a less proportion of alkaline base.

Neither should it be forgotten that a patient living much in the open air and performing much muscular exercise, in order to be "alkalized," requires a smaller quantity of the bicarbonate than the patient who leads a life of inaction. Account must be had, likewise, of the elevation of the temperature, since this favors the alkalization of the economy, in the manner (without doubt) of enforced exercise; and also because it acts upon the nervous system to such a degree that patients find such medication almost intolerable in the hot season.

The author believes it proper to administer the maximum dose which it is proposed to employ, at once and continuously throughout the treatment. This should be in fractional doses, because it is essential to maintain the economy always at the same degree of alkalization. Hence the importance of frequently examining the urine with litmus paper, in order to be assured of the chemical condition of the former, for nothing is so interesting to the physician as a knowledge of the chemical medium in which are accomplished so many of the mysterious morbid phenomena he is called upon to treat.

Claude Bernard recently announced to the French Academy that sugar was a vital, constant and necessary element of the blood. Mailhe adds that it is his conviction that the alkaline bicarbonates constitute a permanent and necessary element, not only of the blood but of the entire economy.

THE CAUSE OF SCORBUTUS.—Gubler. (*La France Médicale, July* 25, 1877.) For a long time it has been generally admitted that the epidemics of scurvy so frequent in distant maritime expeditions result from lack of fresh vegetables. In studying the report of the English commission on the causes of the epidemic of scurvy among the crews of the *Alert* and *Discovery*, M. Gubler was struck by this fact: that the epidemic declared itself with intensity at the moment when the sailors had resumed an active and fatiguing life on land, after a prolonged repose or very moderate labor aboard ship, at the same time that they were absolutely deprived of fresh food, consisting of beef and lime-juice.

For it is to be observed that fresh foods, meat or vegetables, oppose a real obstacle to the development of scurvy, even under the most disadvantageous conditions, and that vegetables do not alone possess prophylactic virtue, although they possess it in a high degree.

The problem propounded by M. Gubler is: what is the rôle of fresh foods? To what principles do they owe the property of protecting the system from the profound alterations which characterize scurvy? Examining the composition of lime-juice, the learned professor remarks that although citric acid predominates, there is also present a certain quantity of mineral substances, and principally potassa, an alkali which exists normally in the animal fluids, and forms one of the indispensable elements of the globules. Lime-juice may then, in a certain measure, take the place of fresh vegetables, by its two principal elements, organic acid and potassa.

These two elements are also found in fresh meat in less proportion; its reaction is acid, and the salts of potassium are present in notable quantity. Salted meats lose their prophylactic virtue because their acidity disappears, and the salts of potassium dissolving in the brine, are replaced by the salts of sodium in excess. This explains the difference in properties, as regards scurvy, which distinguishes fresh from salted meats.

If the theory of M. Gubler is true, and at first sight it is plausible, navigators should provide themselves either with wine charged with its tartar, or with organic salts of potassium, which in small volume represent enormous quantities of fresh vegetable matters. It is an experiment easy to make.

L. W. C.

Morphinism.—A. Spillman.(*Archives Générales de Médecine*, Aug., 1877.) Dr. Levinstein designates under this name a pathological state which has become frequent enough since the vulgarization of subcutaneous injections of morphine by the aid of the Pravatz syringe. The symptomatology of morphinism is about the same as that of alcoholism, even including the delirium. This affection is quite common among the better classes. Patients suffering from it are attacked with trembling, hallucinations, inflammatory phenomena of the lungs and intestines.

Morphine becomes indispensible to those who make use of it to ease the slightest ill-feeling or ennui. But the more they use it the shorter the intervals of calm become.

The three patients whose cases Dr. Levinstein publishes, had become habituated to injections of morphine for several years, and had reached the quantity of more than 15 grains a day. The phenomena observed were: hyperæsthesia, neuralgias, insomnia, anxiety, depression alternating with moments of excitement, muscular contractions, and dryness of the tongue. The intelligence and the memory did not seem to be affected.

When morphine is suppressed, either suddenly or gradually, there follows feebleness, trembling, chills, a pronounced coloration of the face, and abundant perspiration.

The prognosis of morphinism is generally serious enough. The patients soon resort to the means which have procured them relief heretofore, or they succumb to marasmus.

The author cites two cases of patients who committed suicide, and five who have given themselves up to alcoholism.

As regards treatment, the author thinks it impossible to suppress the morphine gradually; it is better to suppress it abruptly and to resort to stimulants, as wine or subcutaneous injections of ammonia, to combat collapse. In all cases the subjects of morphinism should be considered and treated as alienated. Subcutaneous injections of morphine administered by the physician are safe, but in the hands of the patient may become fatal.

Dr. Weinlechner has published a case of a physician who had employed injections of morphine to relieve the pains of a periostitis. The patient had arrived at the point of self-injecting 2½ grammes of morphine a day. His nervous system was considerably shattered, and he was covered with small cutaneous abscesses. He had fallen into a state of complete marasmus.

The subcutaneous injections were suppressed, and replaced by inhalations of chloroform. Recourse was finally had to iodide of potassium, which was followed by almost complete cure.

In a later article, Dr. Levinstein has published a new case of acute poisoning by morphine. It was a lady who had received for the first time an injection of 4½ grs. of morphine. At the end of 20 minutes she had vertigo, was semi-comatose, the pupils were strongly contracted; pulse, 92; respiration, 24. An injection of .0015 grm. of atropine was then given, and repeated twice. The pupils dilated, but the patient fell into a state of somnolence, and the face presented a deep red coloration. Coffee was then given internally, ice applied to the head, leeches to the mastoid processes and in the nostrils. For the somnolence, she was placed in a warm bath. The respiration fell to four in the minute. Electrization of the phrenic nerves was then resorted to. The patient remained completely inert, and fell into a profound sleep. In half an hour the respiration fell to three in the minute. However, the patient awoke for a moment to fall into a profound sleep interrupted by vomiting. The serious results of the poisoning only disappeared at the end of 12 hours, when she recovered consciousness. For 15 days she remained completely prostrated, and only recovered little by little her intelligence and the use of her members.

The urine obtained by the catheter offers the following reactions: treated with soda and sulphate of copper, it presents a blue color ; on heating, the hydrated oxide of copper dissolved in the urine is reduced to the state of insoluble oxide of copper; on adding an alkali and bismuth, a black color is obtained on heating. The angle of polarization is deviated to the right 2.3 deg. At the end of several hours the urine presents all the characters of normal urine.

Dr. Levinstein has completed his studies on morphinism in a recent communication. After having found sugar in the urine of patients suffering from morphinism, he has found albumen. This albuminuria persists for months, even after morphine has been completely suppressed, and when all the other symptoms have disappeared.

All the patients do not become albuminuric; the presence of albumin seems to be in relation with the duration of the use of morphine, and the size of doses employed.

Experiments on animals prove to the author that morphine, given in doses of two to five decigrammes, produces a rapid albuminuria in dogs.

As patients accustomed to the use of morphine injections may seek to deceive the physician, it is well to analyze the urine by Dragendorf's method. One may thus determine the proportion of morphine absorbed. A patient whose urine still contains morphine eight days after the suppression of the drug, must certainly have continued the injections of morphine.

I have been able to prove, in part, the assertions of the author, in a lady of about 50 years who had been advised six years ago to use morphine for the pains of intercostal neuralgia. Since this time the lady had made six to ten injections daily, using thus about 200 grammes in a year. She appears at present like a living skeleton; the appetite is gone, the limbs and head agitated by continual trembling. She is incapable of the least exertion; the least noise agitates her; she avoids the light, and has amblyopia. All means, menaces, persuasion have been employed to induce her to stop this evil habit, which she would only abandon to seek suicide.

The urine of this patient contains a considerable proportion of sugar, but no albumin.

Physicians cannot be too careful in retaining that dangerous instrument, the hypodermic syringe, exclusively in their own hands.

L. W. C.

Case of Chronic Chloral Poisoning.—T. Inglis. (*Edinburgh Med. Journal*, Sept., 1877). A man, aged 47, was admitted to the Royal Edinburgh Asylum with the following history. About seven years ago he was ordered by his physician a mixture containing the hydrate of chloral and the bromide of potassium, in order to relieve a spasmodic retention of urine. He took about a drachm of each of these drugs daily for six years regularly, and during this time neither he nor his friends observed any hurtful effect. But the drug so enslaved him that he had a craving for its sedative effect. Six years afterwards he had an attack of bronchitis, and took chloral to allay the breathlessness and procure sleep. The recovery was rapid, but business care brought on some mental depression, and he sought oblivion in choral. At first a 60-grain dose was taken, but was gradually increased till he took 180 grains per diem. He carried a bottle with solution of chloral in his pocket and took a dose of ten grains every hour, or every half-hour; and if he chanced to waken at night he repeated the dose. Sleep was not induced, but a soothing feeling. He complained of no headache, vertigo or depression as the result of the drug, but a feeling of lassitude and nervous debility and exhaustion arose, together with an inaptitude for work and incapacity for continuous thought. His appetite declined, food lost its relish; nausea and sour eructations were complained of, and vomiting occurred frequently. He had slight jaundice; the fæces were hard and white.

As he abandoned chloral eating, he became untruthful, deceitful, irritable and passionate. The natural affection for his wife and children became blunted; he lost his regard of duty and self-respect. When he discontinued using the chloral he took to whisky. He did not take enough to produce complete intoxication, but sufficient to keep him in a chronic state of confusion. He became violent and unmanageable, and threatened suicide. Diarrhœa set in, and was followed by a great discharge of blood from the bowels. Then he got into a state of delirium tremens, which terminated by three severe epileptiform attacks following each other at intervals of four hours.

On admission he appeared prematurely old and broken-down; he was perfectly silly and childish, and almost imbecile in manner. He would laugh and cry alternately without adequate cause ; took no interest in what was going on around him ; his memory, almost obliterated ; his answers, incoherent. There was persistent muscular tremulousness of the upper and lower extremities ; power of co-ordination was lost, and he was unable to walk alone. The tongue was furred in the centre, tremulous and pointed markedly to the right side. Articulation was impaired. The pupils were dilated, irregular at the margins and insensible to light. The right side of the face was partially paralysed; the reflex action of the cord was much impaired. He complained of restlessness and exhaustion, but had no headache or neuralgic pains. Respiration and circulation normal. No albumen, sugar, bile or tube casts in urine.

No chloral or any narcotic was given after admission. Was ordered a tonic mixture containing strychnine, strengthening diet, and as much exercise in the open air as he could bear. The regular action of the bowels was promoted by a gentle aperient. The appetite for food returned slowly, but he gained in flesh and appearance very rapidly. The tremulousness and paralytic symptoms disappeared in an astonishingly short time. Pupils remained dilated for about three weeks, but their outlines became regular, and they contracted normally under the influence of light in a few days. Mentally his convalescence was equally speedy. Memory and coherent thought soon returned. After a short stage of stupor his intellect regained strength by degrees, and his emotions and affections resumed their natural condition. He was discharged three months after admission.

